# Harnessing hybrid buses in the near term leads to faster transit decarbonization

**DOI:** 10.1016/j.isci.2025.113567

**Published:** 2025-09-13

**Authors:** Mahsa Arabi, Tolu Oke, Erin Baker, Jimi Oke

**Affiliations:** 1 Department of Civil and Environmental Engineering, University of Massachusetts Amherst, Amherst, MA, USA; 2 Department of Mechanical and Industrial Engineering, University of Massachusetts Amherst, Amherst, MA, USA

**Keywords:** Applied sciences, Electrical engineering, Electrical system

## Abstract

To achieve net-zero emissions, transit systems will eventually have to transition to fully electric buses. We introduce an integrated decision-making and energy modeling framework to analyze transition strategies from diesel to zero-emitting buses under various budget scenarios. Using a detailed energy model of the buses to predict fuel usage, we then optimally assign types of buses to scheduled trips to minimize diesel consumption and emissions. Applying this framework to the Pioneer Valley Transit Authority network, we find that under realistic budget and infrastructure constraints, an electrification future that allows a mix of electric and hybrid buses reduces emissions more effectively than an all-electric future. Specifically, for a fixed budget, incorporating hybrid buses results in an additional 6% emissions reduction over the 18-year planning horizon compared to an electric-only future. This shows that hybrid buses can speed up the elimination of diesel buses and maximize emissions reduction while mitigating infrastructure and financial constraints. Our framework evaluates different fleet transition futures and provides transit agencies with a data-driven framework to plan investments and meet their decarbonization targets under realistic budgetary and infrastructure constraints.

## Highlights

• Purchasing hybrids along with electric buses leads to the earlier phase-out of diesel buses
• Trip-level energy and optimization models inform route and fleet deployment decisions
• Findings are robust to assumptions on charging capacity and vehicle range

## Graphical abstract

**Figure:**
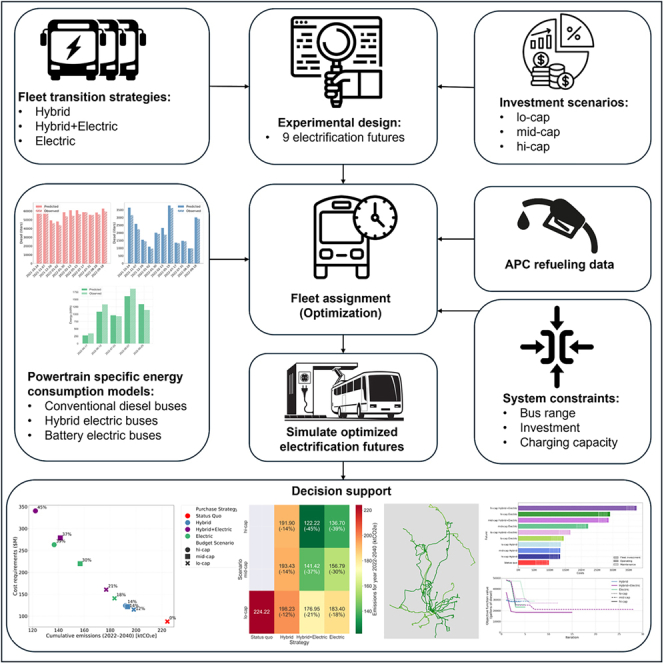

## Introduction

As the world faces an increasing climate crisis, mitigating emissions from transportation has become an important policy and research priority.^1^^,^^2^^,^^3^^,^^4^^,^^5^ Globally, the transportation sector accounted for approximately one-seventh of greenhouse gas (GHG) emissions in 2023.^6^ In the United States, it accounted for nearly a third of domestic emissions.^7^ While passenger vehicles are the most emissions-intensive mode in the transportation sector (0.13 kgCO_2_ per passenger-kilometer), conventional diesel buses closely follow, emitting 0.12 kgCO_2_ per passenger-kilometer—more than air travel, passenger railroads, and rail transit.^8^ Electrification and other alternative-fuel pathways are widely considered key strategies for decarbonization.^9^^,^^10^^,^^11^^,^^12^^,^^13^ For example, the transition from diesel (Throughout this article, we refer to “conventional diesel” as simply “diesel” buses for the sake of brevity) to an all-electric (Similarly, we refer to “battery-electric” as simply “electric” buses for brevity) bus fleet in Los Angeles has yielded environmental benefits equivalent to approximately $65 million in annual savings.^14^ However, the challenge remains: how can transit agencies transition to net-zero emissions while constrained by investment and charging infrastructure?

An analysis of fleet transitions indicates significant CO_2_ reduction potential when replacing diesel buses with electric alternatives, particularly when aligned with shifts toward renewable energy in grid electricity.^15^ Cost–benefit assessments of early-stage fleet electrification have demonstrated that targeted investments in electric buses can maximize environmental benefits while remaining within financial constraints.^16^ Electric buses offer zero tailpipe emissions, making them an attractive solution for reducing urban air pollution and its associated health impacts, such as cardiovascular and respiratory diseases.^17^ Additionally, electric buses contribute to quieter urban environments compared to diesel buses.^18^ However, the high upfront costs of electric buses and the required charging infrastructure present barriers to large-scale adoption.^19^ These initial costs result in electric buses having 7% higher life cycle costs than diesel and hybrid (Similarly, we refer to “hybrid-electric” as “hybrid” buses) buses.^20^ However, as battery costs continue to decline, the long-term investment in electric buses should become more attractive.^21^ Beyond financial barriers, however, infrastructure upgrades present additional challenges, as they require substantial planning for integration into existing transit networks to ensure the electrical grid can support increased demand.^22^^,^^23^

Hybrid buses, though understudied and often dismissed,^24^ can play an important role in the mid-term transition. Compared to diesel, hybrid buses offer a 40% reduction in CO_2_ life cycle emissions and a 30% increase in driving range, meaning they can travel longer distances before requiring refueling.^25^^,^^26^ This extended range reduces downtime for refueling, enhances operational efficiency, and allows greater route flexibility. While hybrid buses do not achieve the zero tailpipe emissions of electric buses, they present a practical solution due to their lower initial capital costs and infrastructure requirements. Most importantly, they can be deployed rapidly, as they do not require changes to existing charging infrastructure.

Recent research has largely focused on maximizing the operational efficiency of electric buses, including charging infrastructure placement,^27^^,^^28^ battery sizing,^29^ and advanced charging technologies.^30^ Other studies have emphasized the economic and environmental benefits of electric bus adoption, using methods such as life cycle assessments, techno-economic evaluations, and Geographic Information System (GIS)-based analyses.^21^^,^^31^^,^^32^ Some recent work has also developed multi-phase optimization models to support incremental bus fleet electrification under real-world constraints.^33^ Effective fleet electrification has been shown to depend not only on charging infrastructure but also on strategic combinations of battery capacity and charging power, highlighting the need for integrated long-term planning approaches.^34^^,^^35^ Studies in various urban contexts have demonstrated that public transport electrification can lead to substantial emissions reductions and health benefits, with estimates indicating a 75% decrease in pollutants and significant public health savings in Delhi.^36^ Similarly, financial analyses have emphasized the role of targeted subsidies and investment timing in accelerating electrification efforts, as observed in Milan’s transition planning and Singapore’s policy-driven adoption timeline.^37^^,^^38^

However, few studies have explicitly addressed transition strategies—specifically, questions surrounding the optimal rate at which transit agencies should adopt electric buses, the role of hybrid buses, and how specific routes should be prioritized for electrification. Even fewer studies have analyzed how these vehicles should be deployed within transit networks to maximize emissions reductions while minimizing system costs. These are critical gaps, as strategic route-level decisions can significantly impact the feasibility and efficiency of the transition.

Another particular limitation of existing research is the lack of accurate energy modeling approaches tailored to transit fleet transitions. Energy modeling has evolved significantly. Early models focused on diesel fuel consumption.^39^ Later works developed concave and convex hybrid bus fuel consumption models.^40^^,^^41^ More recent models account for electric vehicle and regenerative braking efficiency.^42^^,^^43^ More recently, data-driven machine learning approaches have been applied to transit energy modeling with strong predictive capabilities.^44^ Tractive models offer more precise energy estimates incorporating real-world vehicle dynamics, including grade, acceleration, and regenerative braking efficiency. This level of detail is essential for optimizing fleet transitions at the trip level, yet has been largely absent from prior research. However, no prior studies have used tractive multi-powertrain energy models to analyze fleet transitions.

This article makes three key contributions toward addressing these gaps. First, we present an optimization framework for fleet transitions under investment constraints, balancing battery electric buses, hybrid electric buses, and conventional diesel buses to minimize emissions and fuel consumption over the planning horizon. Second, we incorporate a tractive multi-powertrain energy model to provide detailed energy predictions, capturing real-world vehicle dynamics such as grade, acceleration, and regenerative braking efficiency. Third, we create a strategic electrification planning framework by simulating ten transition pathways to help transit agencies evaluate trade-offs between cost, energy consumption, and emissions reduction goals at the route level. This approach provides a data-driven decision support tool and helps transit agencies, stakeholders, and researchers to identify and evaluate electrification transition pathways to balance financial constraints with emissions reduction targets and ensure a sustainable shift toward cleaner transit.

### Objectives and framework

**Figure 1:**
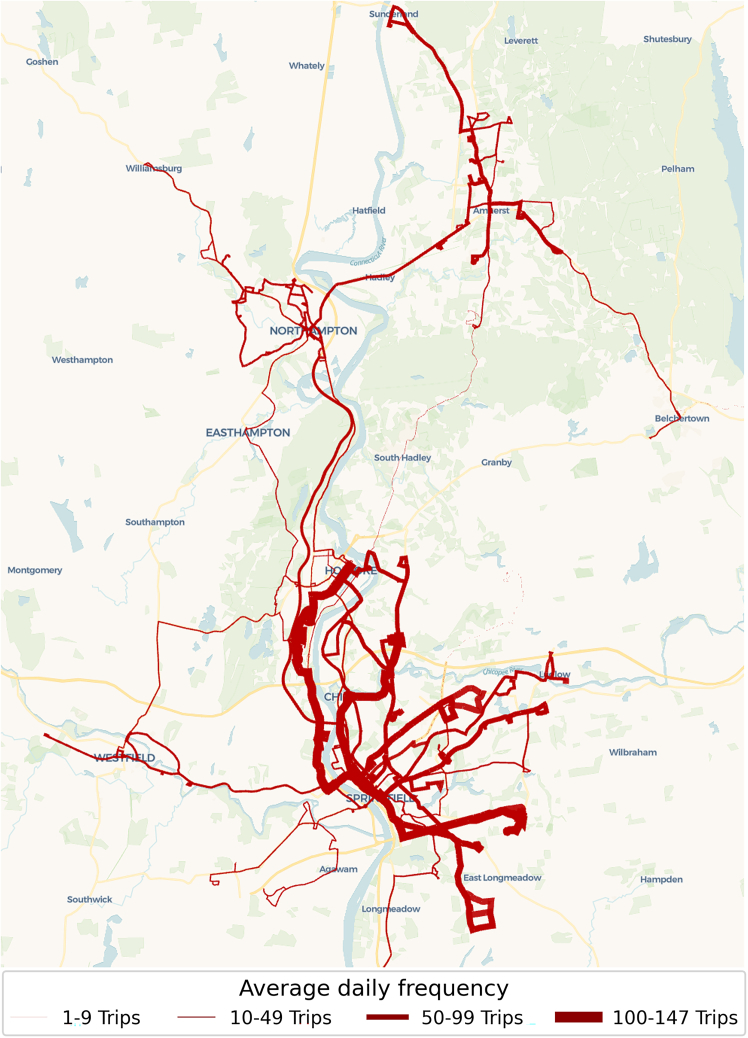
Case study: the Pioneer Valley Transit Agency (PVTA) bus network

**Figure 2:**
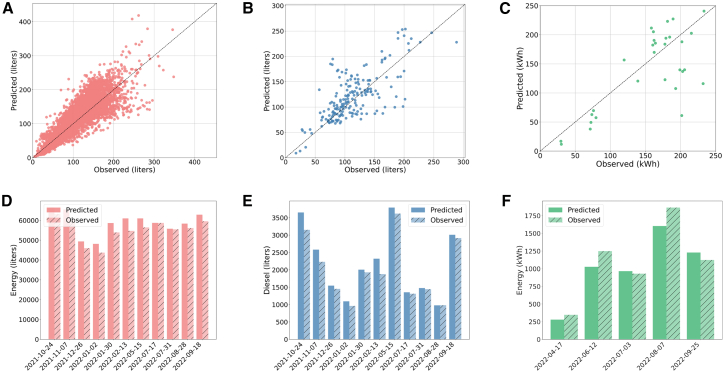
Vehicle- and system-level validation of energy submodels across powertrain types over the test period (A–C) Refueling-interval vehicle-level predicted versus observed energy consumption for (A) diesel, (B) hybrid, and (C) electric buses. (D–F) Weekly system-level energy consumption for (D) diesel, (E) hybrid, and (F) electric buses.

In this article, we investigate the conditions under which hybrid and electric buses both play an important role in the transition toward net-zero transit emissions, using the Pioneer Valley Transit bus network in the US state of Massachusetts as a case study (Figure 1). Our optimization framework integrates a tractive multi-powertrain energy model—a novel application for this kind of effort—calibrated to provide accurate energy predictions per trip (Energy model validation results are shown in Figure 2). Ultimately, the framework assigns a powertrain type (diesel, hybrid, or electric) to each scheduled trip with the objective of minimizing cumulative diesel consumption and emissions over an 18-year planning horizon (through 2040) while incorporating key constraints such as demand fulfillment, annual investment limits on vehicle procurement, and the gradual expansion of charging infrastructure.

We examine three fleet purchase strategies: new hybrid only (*Hybrid*), new electric only (*Electric*), and a mix of both (*Hybrid+Electric*). We also consider three annual budget-constrained scenarios: $10M (*lo-cap*), $20M (*mid-cap*), and $30M (*hi-cap*). Agencies typically allocate capital budgets annually, and consistent yearly caps enable a more realistic representation of procurement and deployment planning over time. Furthermore, we account for only the investment costs of new bus purchases and not those of infrastructure expansion. Instead, we explicitly constrain the growth of charging infrastructure to reflect realistic deployment limitations, including utility coordination, site availability, grid interconnection, and capacity constraints. These factors are largely external to the transit agency, making infrastructure expansion an exogenous constraint in our model. Optimizing each of the strategies under each budget scenario yields nine electrification futures, which we evaluate in terms of energy consumption, well-to-wheel (WTW) emissions reduction, costs, and fleet composition. Additionally, we consider the *Status Quo*, reflecting the current fleet composition of the network, which remains unchanged throughout the planning horizon. The futures are summarized in Table 1. Further details on the study network, energy modeling and optimization methods are provided in STAR Methods.

**Table 1:** Matrix of evaluated futures

Purchase Strategy	Description	Budget scenario (annual)		
		*lo-cap ($10M)*	*mid-cap ($20M)*	*hi-cap ($30M)*
*Status Quo*	Maintain existing fleet composition	✓	–	–
*Hybrid*	All new buses are hybrid	✓	✓	✓
*Hybrid+Electric*	New buses can be either hybrid or electric	✓	✓	✓
*Electric*	All new buses are electric	✓	✓	✓

## Results

### Hybrid buses allow quicker elimination of diesel buses

**Figure 3:**
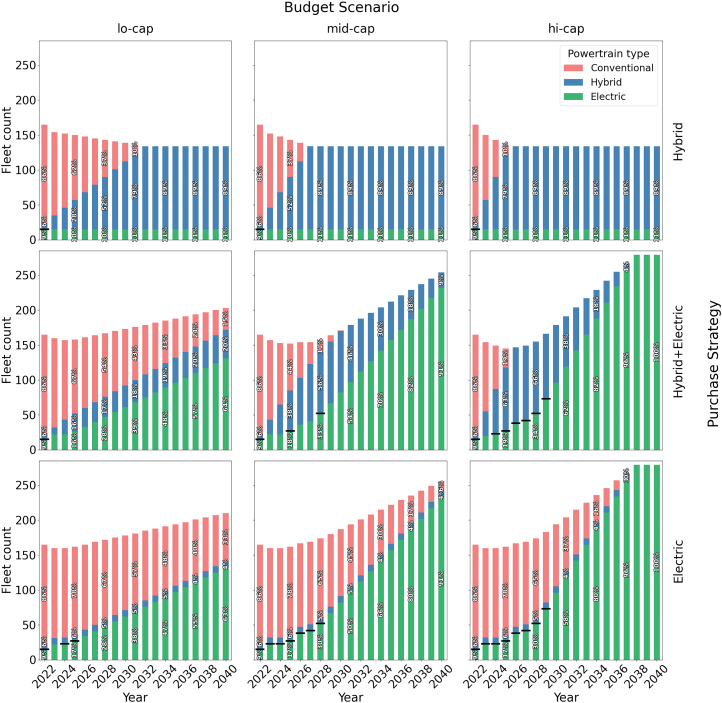
Powertrain distribution across simulated futures Fleet counts are shown as stacked bars, with percentage annotations every three years. Black horizontal lines are shown only when the charging infrastructure is constrained.

Electrification futures that incorporate hybrid buses enable transit agencies to accelerate the retirement of diesel buses, especially in the early years of the transition. As shown in Figure 3, two of the hi-cap scenarios (*Electric* and *Hybrid+Electric*) reach zero tailpipe emissions by 2038. The path to get there is quite different, however: the *hi-cap Hybrid+Electric* future eliminates all diesel buses by 2027, compared to the *hi-cap Electric* future, which still retains 118 diesel buses in the same year due to budget and charging infrastructure constraints. In fact, the *hi-cap Electric* future contains diesel buses for 10 additional years, until 2037. This contrast illustrates the value of hybrid buses as a transitional strategy. While not emissions-free, they allow earlier life cycle reductions during times when infrastructure capacity restricts full electrification.

An important driver is the charging infrastructure, which is modeled as an exogenous constraint, since it is shaped by factors such as utility coordination, space availability, and grid capacity. In the early years, limited availability prevented widespread electric bus deployment, and the model will assign hybrid buses, when available, to reduce diesel reliance. As shown by the black horizontal lines in Figure 3, limited charging availability initially delays electric bus deployment in the *hi-cap Electric* future, forcing continued diesel use. In later years, annual budget caps became the main constraint, slowing fleet expansion even when cumulative funding is sufficient. This staggered investment pattern amplifies the effects of early infrastructure bottlenecks, particularly in the first half of the transition. We present detailed sensitivity analyses of both charging infrastructure and electric bus range later in this section.

The reduction in active hybrid bus deployment in later years, particularly in the *hi-cap Hybrid+Electric* future, reflects a strategic shift toward electric buses as infrastructure expands. These hybrid buses remain in the fleet as reserve capacity, helping maintain service continuity when electric buses face charging, range, or reliability challenges. Maintaining hybrids as a backup mitigates early reliability risks and provides operational flexibility during the transition. This practice aligns with real-world fleet management, where agencies retain reserve vehicles to manage uncertainties when adopting new technologies.^45^

### *Hybrid+Electric* futures minimize cumulative emissions in all scenarios

**Figure 4:**
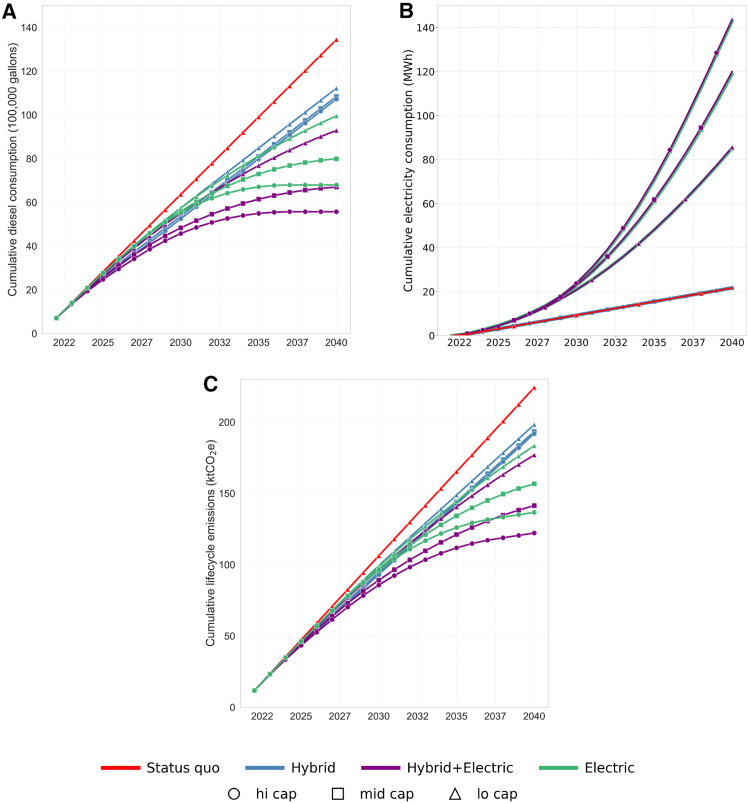
Energy use and emissions across simulated futures (A) Cumulative diesel consumption, (B) cumulative electricity consumption, and (C) life cycle emissions over the 18-year planning horizon for the optimized futures.

*Hybrid+Electric* futures, which combine hybrid and electric buses, achieve greater reductions in both diesel use and emissions compared to hybrid-only or electric-only strategies. Cumulative diesel consumption and associated emissions across all futures over the planning horizon are shown in Figure 4. Among all futures, the *hi-cap Hybrid+Electric* future yields the greatest cumulative reduction, lowering diesel consumption by approximately 59% (Figure 4A) and emissions by 45% (Figure 4C) relative to the *Status Quo*. In this future, early diesel savings will be achieved by substituting diesel buses with hybrids when charging capacity constraints limit the deployment of electric buses. Figure 4B presents cumulative electricity consumption across futures. The *Status Quo* and all *Hybrid* futures show identical electricity consumption trends, since their electric bus deployment remains unchanged throughout the simulation horizon. Similarly, the *Hybrid+Electric* and *Electric* futures exhibit nearly identical electricity consumption, reflecting their comparable electric bus fleet sizes.

The *mid-cap Hybrid+Electric* and *hi-cap Electric* futures also achieve notable reductions. In the former, diesel consumption and emissions are reduced by approximately 50% and 37%, respectively, while in the latter, diesel consumption is reduced by 49% and emissions by 39%. While the *mid-cap Hybrid+Electric* future results in slightly lower diesel use than the *hi-cap Electric* future, its emissions are slightly higher. This is due to the non-linear relationship between diesel consumption and emissions. Rather than applying a fixed emissions-per-liter factor, we account for vehicle fuel economy and powertrain-specific emission rates per km, as shown in Equation 20 and Equation 21. As a result, even a smaller volume of diesel or hybrid fuel use can result in relatively higher emissions depending on how trips are distributed across vehicle types and trip distances. These results highlight the importance of adopting both hybrid and electric buses in achieving early and sustained emissions reductions when infrastructure expansion is constrained. In addition to system-wide estimates, our framework enables analysis of emissions at the route level, allowing agencies to identify corridors with the greatest potential for improvement in each future (See Figure S1 for more details.)

### Deeper emissions reductions in the *hi-cap Hybrid+Electric* future require substantially higher costs

**Figure 5:**
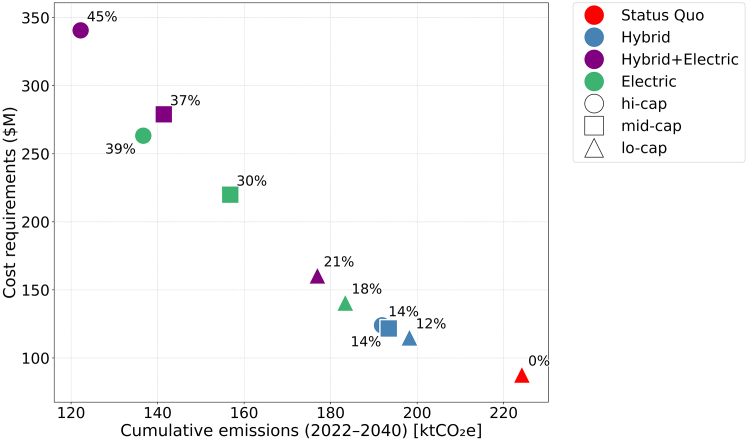
Trade-offs between cumulative emissions (2022–2040) and total cost, including fleet investment, maintenance, and operating costs for simulated futures Annotations indicate the percentage cumulative emissions reductions in each future relative to the *Status Quo*.

There is a trade-off between cumulative emissions reduction and cost. Futures that achieve the greatest decarbonization—*hi-cap Hybrid+Electric*, *mid-cap Hybrid+Electric*, and *hi-cap Electric*—have the highest cumulative costs by 2040 (Figure 5) (The breakdown of cumulative costs is shown in Figure S2). Among these, the *hi-cap Hybrid+Electric* future is the most expensive, with a cumulative net present value (NPV) of $340 million, reflecting the simultaneous deployment of hybrid and electric buses. The high costs of the *Hybrid+Electric* futures are related to the early retirement of hybrids in some cases. In the *mid-cap* and *hi-cap Hybrid+Electric* futures, the optimization model occasionally replaces hybrid with electric buses before the end of their life. This reflects a realistic situation where, once charging infrastructure limitations (Constraint ((4)), visualized in Figure S3) are no longer binding, transit agencies may choose to replace hybrid buses with electric buses to reduce emissions further. Fleet composition data (Figure 3) show that in the *hi-cap Hybrid+Electric* future, hybrid buses grow from about 6% of the fleet in 2022 to 66% by 2028, when diesel buses are phased out. Over time, many of these hybrids are replaced with electrics. Rather than being prematurely retired, they are retained as reserve vehicles, consistent with real-world fleet management practices.^45^ This approach enables maximizing emissions reductions while maintaining operational flexibility.

Futures located on the Pareto frontier (those tending toward the lower-left region of the plot) represent the most efficient trade-offs between cost and emissions reduction. We note that the *mid-cap Hybrid+Electric* future is (slightly) Pareto-dominated as it is both more expensive and results in higher cumulative emissions than the *hi-cap Electric* future. This is driven by annual budget constraints. In *mid-cap Hybrid+Electric*, the model initially invests in hybrid buses to reduce diesel use while charging infrastructure is still limited. However, as infrastructure expands in later years, these hybrids are partially replaced with electric buses before the end of their useful life, increasing total costs without proportional emissions benefits. In contrast, the *hi-cap Electric* future can sustain a consistent investment in electric buses once infrastructure becomes available, avoiding early hybrid investments and reducing cumulative costs. This highlights how annual budget caps can create inefficiencies by limiting flexibility, even when total funding is sufficient.

### The *hi-cap Hybrid+Electric* future remains robust under varying electric bus range and annual charging capacity assumptions

**Figure 6:**
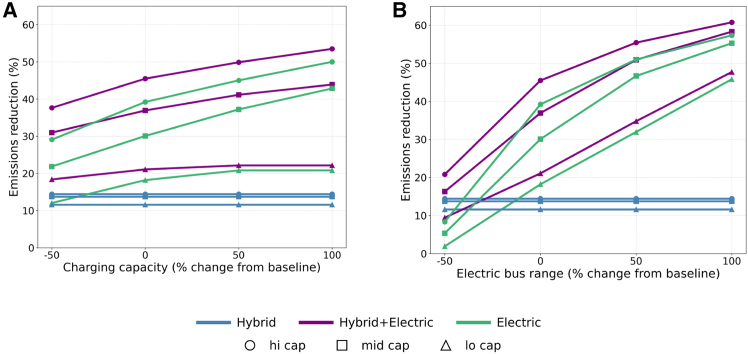
Emissions sensitivity to charging and range Sensitivity analysis of emissions reductions (%) under variations in (A) charging capacity and (B) electric bus range across different fleet transition strategies. The baseline daily charging capacity reflects year-specific growth, starting at 23 buses per day in 2023 and increasing annually to reflect infrastructure expansion (Figure S3). After 2028, capacity is assumed to grow by 40% per year through 2040. The baseline electric bus range is set at 113 km (km).

We investigate how assumptions about charging capacity affect the emissions reductions of simulated futures (See Figure S3 for the summary of baseline assumptions.) As charging capacity increases, emissions decrease across all futures that incorporate electric buses (Figure 6A). However, these changes have only a minor effect on the relative attractiveness of different futures. A 100% increase in charging capacity allows the *mid-cap Electric* future to perform comparably to the *mid-cap Hybrid+Electric* future, with both achieving approximately 40% emissions reduction relative to the *Status Quo*. The *hi-cap Electric* future still performs worse than the *hi-cap Hybrid+Electric* future. This suggests that charging infrastructure growth is more influential under mid-level budget constraints.

We also find that the electric bus range has a greater impact on overall emissions reductions compared to the charging capacity (Figure 6B). A 100% increase from the base electric range of 113 km improves the cumulative emissions reduction of *hi-cap Hybrid+Electric* by about 15 percentage points (pp). Furthermore, a 100% increase in range enables the *mid-cap Hybrid+Electric* future to outperform the hi-cap Electric future (by 1 pp). This is because with extended range, fewer electric buses are needed to cover long trips, which allows the model to assign hybrids more strategically to higher-impact routes, increasing overall emissions savings in the *Hybrid+Electric* futures. Conversely, a 50% reduction in electric bus range significantly worsens the performance of all three *Electric* futures, to the point where they become less effective than *Hybrid*-only futures. Most significantly, the emissions reduction in *hi-cap Electric* (the greatest proportion of electric buses) worsens by about 30 pp, further highlighting how crucial bus range is to the sustainability of electric-only strategies. In contrast, the *hi-cap Hybrid+Electric* future consistently achieves the highest emissions reductions across the full range of sensitivity assumptions.

In general, we find that the *hi-cap Hybrid+Electric* future remains robust across a wide range (from −50% to +100%) of assumptions regarding both charging infrastructure and electric bus range. This further indicates that hybrid buses cannot be ignored as agencies navigate short-term uncertainty and ramp-up times for infrastructure and battery technology development.

## Discussion

We developed a high-level decision support framework to optimize the transition from conventional diesel buses to zero-emission alternatives between 2022 and 2040, accounting for realistic budget constraints, charging infrastructure limitations, and vehicle range assumptions. Applying this framework to the Pioneer Valley Transit Authority (PVTA), we integrated a tractive multi-powertrain energy model for diesel, hybrid, and electric buses, calibrated to the specific characteristics of the PVTA fleet. This model predicts energy consumption by incorporating variables such as grade, passenger load, and road conditions, providing a more dynamic and accurate representation compared to studies that rely on fixed values. We evaluated four transition strategies: *Status Quo* (no change in current composition); *Hybrid* (new fleet restricted to hybrid buses); *Hybrid+Electric* (both hybrid and electric buses can be purchased); and *Electric* (new fleet restricted to electric buses), alongside three fleet budget scenarios (*lo-cap*, *mid-cap*, *hi-cap*), resulting in ten electrification futures. For each future, we solved for the optimal powertrain assignment, and thus fleet composition, and assessed each of them based on fleet composition, diesel consumption, life cycle emissions reduction, and costs. Our emissions analysis incorporates both upstream (well-to-tank) and downstream (tank-to-wheel) emissions for all powertrain types, including emissions from electricity generation, providing a comprehensive life cycle perspective.

Our findings highlight the critical role of hybrid buses as a transitional technology. We found that an electrification pathway that allows for both electric and hybrid buses results in the highest emission reductions. Under high-budget scenarios, the *Hybrid+Electric* strategy reduces life cycle (well-to-wheel) emissions by up to 45% over an 18-year horizon compared to the *Status Quo*, and by 6% compared to an *Electric*-only strategy. This strategy also enables the complete phase-out of diesel buses approximately 10 years earlier than the all-electric strategy, when charging infrastructure expansion is limited. For mid-sized transit agencies such as PVTA, a phased transition provides a practical and effective pathway to decarbonization. The transition starts by investing in hybrid buses in areas with limited charging infrastructure, then gradually expanding the use of battery electric buses as additional capacity and funding become available.

We also conducted sensitivity analyses on charging capacity growth and electric bus range assumptions. We found that changes in these key parameters did not change the results; The *hybrid + electric* future consistently outperforms other strategies in terms of emissions reduction. Future research could extend the framework by evaluating alternative charging strategies, such as depot versus opportunity charging, modeling en-route charging options, and incorporating infrastructure deployment costs. Emissions modeling could also be refined to reflect dynamic grid emissions and seasonal energy mixes, capturing temporal variations in upstream emissions. Additionally, the framework can support equity-focused planning by incorporating socioeconomic and demographic factors associated with different routes and their localized emissions. Overall, this decision support framework provides transit planners and policymakers with a flexible tool to evaluate electrification strategies, support infrastructure investment decisions, and guide sustainable fleet transitions.

### Limitations of the study

This study presents a practical framework to support transit agencies in planning their fleet electrification strategies. However, several modeling assumptions should be noted. The optimization model is designed as a high-level decision support tool that assigns powertrain types to trips, rather than tracking individual vehicle-level assignments. The required number of buses from each powertrain type is estimated based on cumulative daily vehicle kilometers and range constraints, providing a practical estimate without modeling detailed vehicle dispatching. The required number of buses for each powertrain type is estimated based on daily vehicle kilometers and range constraints, rather than detailed vehicle dispatch. Charging infrastructure availability is modeled as an exogenous constraint, but infrastructure costs themselves are not explicitly considered. Additionally, charging sessions are assumed to occur overnight, consistent with current operational practices in the case study agency.

We also used regional average electricity emissions factors without accounting for hourly or seasonal changes in the grid. This simplification helps keep the approach practical and broadly applicable, while still providing valuable insights for transit planning. Future work can extend this framework to include detailed vehicle dispatch models and dynamic grid emissions. Finally, our energy model captures key operational factors, including speed, acceleration, grade, and passenger load, when estimating diesel usage. However, we convert diesel consumption to emissions using a linear relationship based on standard life cycle factors. This is a common approach, but it might not fully capture the non-linear effects from engine load, cold starts, or other operating conditions. Future work could consider more detailed emissions models to estimate life cycle GHG emissions.

## Resource availability

### Lead contact

Requests for further information and resources should be directed to and will be fulfilled by the Lead Contact, Mahsa Arabi (marabi@umass.edu).

### Materials availability

This study did not generate new unique materials.

### Data and code availability

All the data and code used in generating the results and figures in this article are publicly available at https://github.com/narslab/transit-electrification-futures.git.

## Acknowledgments

This research was supported by the Pioneer Valley Transit Authority and by ELEVATE, which is funded by the National Science Foundation (NSF) Research Traineeship (NRT) program (Award Number 2021693). We also acknowledge partial support from the NSF Expeditions in Computing project CoDec (Award CNS-2325956) and from the U.S. Department of Energy (DE-EE0010143). The authors thank Sandra Sheehan and Alex Forrest from the Pioneer Valley Transit Authority for supporting this research and providing valuable insight and feedback on the study. We also thank Glenn Barrington, Connie Englert, Tom Vincent, Matt Moretti (University of Massachusetts Transit Service), Nicole M. Rohan, and Jonathan McHatton (Springfield Area Transit Company) for providing operations data and insights for the study.

## Author contributions

Conceptualization, MA, TO, and JO; methodology, MA, and JO; investigation, MA, TO, EDB, and JO; writing-–original draft, MA; writing-–review & editing, MA, TO, EDB, and JO; funding acquisition, TO, EDB, and JO; resources, MA, TO, and JO; supervision, EDB, and JO.

## Declaration of interests

The authors declare no competing interests.

## STAR★Methods

### Key resources table

**Table:** 

REAGENT or RESOURCE	SOURCE	IDENTIFIER
**Deposited data**		

Datasets and figure source data	PVTA & This paper	https://github.com/narslab/transit-electrification-futures.git
Greenhouse gas emission factors	EPA GHG Inventories	https://www.epa.gov/system/files/documents/2024-02/ghg-emission-factors-hub-2024.pdf

**Software and algorithms**		

Python	Python Software Foundation	https://www.python.org
Gurobi Optimizer (gurobipy)	Gurobi Optimization, LLC	https://www.gurobi.com
Energy & optimization code	This paper	https://github.com/narslab/transit-electrification-futures.git

### Method details

This section describes our approach to evaluating optimal medium-term fleet electrification strategies using the Pioneer Valley Transit Authority (PVTA) bus transit network as a case study.^46^ First, we computed bus trip trajectories using operations data from Automatic Passenger Counters (APC). Then, we developed a system-wide energy model by calibrating submodels specific to each powertrain type: diesel, hybrid, and electric. Following this, we formulated a constrained optimization problem for assigning powertrains to bus trips in order to minimize diesel consumption predicted by the energy model. We then designed three bus fleet transition strategies and three budget scenarios, which, together with the *Status Quo*, resulted in ten futures, which were evaluated in terms of fleet composition, diesel consumption, emissions, and costs. A comprehensive list of abbreviations, as well as the parameters, variables, indices, and sets incorporated into the energy model and optimization frameworks, is summarized in the following tables.

**Table:** Glossary of abbreviations and units

Abbreviation	Description
APC	Automatic Passenger Counter
CDF	Cumulative Distribution Functions
Diesel	Conventional Diesel Bus
Electric	Battery Electric Bus
GHG	Greenhouse Gas
GIS	Geographic Information System
Hybrid	Hybrid Electric Bus
Km	Kilometer
L	Liter
MAE	Mean Absolute Error
MAPE	Mean Absolute Percentage Error
MdAPE	Median Absolute Percentage Error
MSA	Metropolitan statistical areas
NPV	Net Present Value
PVTA	Pioneer Valley Transit Authority
RMSE	Root Mean Squared Error
TTW	Tank-To-Wheel
WTT	Well-To-Tank
WTW	Well-To-Wheel

**Table:** Parameters used in energy model and optimization frameworks

Symbol	Description	Value
*A*_*p*_	Frontal area (m^2^)	{*C*: 6.99, *H*: 7.44, *B*: 7.44}
*C*_*D*_	Drag coefficient	0.78
*C*_*r*1_	Rolling resistance coefficient	1.476·10^−4^
*C*_*r*2_	Rolling resistance coefficient	5.719·10^−3^
$C_{H_{i}}$	Altitude correction factor	1-0.085*H*_*i*_
*cost*_*p*_	Purchase cost of bus with powertrain *p* (M$)	{*C*: 0.4, *H*: 0.9, *B*: 1.3}
*η*_*d*,*p*_	Driveline efficiency	{*C*: 0.94, *H*: 0.94}
*g*	Acceleration due to gravity (m/s^2^)	9.81
*M*^*inv*^	The maximum yearly investment (M$)	{lo-cap: 10, mid-cap: 20, hi-cap: 30}
*M*^*flt*^	Maximum fleet size	1000
$M_{y}^{cap}$	Maximum daily number of buses that can be charged	Figure S3
*r*_*p*_	Range of a bus powertrain *p* (km)	{*C*: 174, *H*: 201, *B*: 113}
*Ρ*	Air density (kg/m^3^)	1.23

**Table:** Variables, indices, and sets used in energy model and optimization frameworks

Symbol	Description
*a*_*i*,*t*_	Acceleration of bus *i* (m/*s*^2^) at timestamp *t*
*i*∈[1,*N*_*p*_]	Index of bus
*d*_*τ*_	Trip *τ* distance (km)
*D*_*p*,*i*,*t*_	Diesel consumption of bus *i* with powertrain type *p*∈{*C*,*H*} at timestamp *t*
*eco*_*p*_	The fuel economy of powertrain *p*
*E*_*i*,*t*_	Electrical energy consumption of BEB *i* (kWh) at timestamp *t*
*G*_*i*,*t*_	Road grade (tangent) at the location of bus *i* at timestamp *t*
*m*_*i*,*t*_	Mass of bus *i* including passengers (kg) at timestamp *t*
$M_{y}^{TTW}$	tank-to-wheel emissions in year *y* (gCO_2_ e)
$M_{y}^{WTT}$	well-to-tank emissions in year *y* (gCO_2_ e)
$M_{y}^{WTW}$	well-to-wheel emissions in year *y* (gCO_2_ e)
*N*_*p*,*y*_	Number of fleet of powertrain *p* in year *y*
*p*∈{*C*,*H*,*B*}	Powertrain type: {*C*: *diesel*, *H*: *hybrid*, *B*: *electric*}
$r_{i}\in \left[1,N_{r_{i}}\right]$	Refueling ticket interval index in observation dataset
τ∈T	Set of trips
*v*_*i*,*t*_	Speed of bus *i* (m/s) at timestamp *t*
*VKT*_*p*,*y*_	The total annual distance (km) traveled by powertrain type *p* in year *y*
*x*_*p*,*τ*,*y*_∈{0,1}	Bus assignment indicator
y∈Y	Set of years in the planning horizon

#### Data

We used operational data from October 2021 to September 2022, provided by the Pioneer Valley Transit Authority (PVTA), including Automatic Passenger Counter (APC) records and refueling logs. The APC data captured the timestamp and location of each stop event, along with the number of passengers boarding and alighting. Although APC records are event-based and primarily reflect stationary times, we used them to estimate average speeds and accelerations between stops by calculating time intervals between consecutive events. These estimates, when calibrated, aligned well with observed system-wide and vehicle-level performance. In the absence of comprehensive drive cycle data, this approach offered a practical alternative. We also obtained refueling data, including timestamps and fuel volumes, and bus fleet characteristics such as vehicle age, powertrain type, and cumulative kilometers.

PVTA is the second-largest transit agency in Massachusetts, serving an area of over 1554 square km in Hampden and Hampshire counties in western Massachusetts. In 2022, the agency operated 213 buses on 42 fixed routes, as well as 134 vans that provide services under the Americans with Disabilities Act (ADA) and senior services. For the purposes of this study, we focused only on the fixed route service. The average number of daily trips across the PVTA network is 1,705, with the cumulative daily distance traveled by all buses amounting to 14,502 km. PVTA’s fixed-route fleet in 2022 comprised three powertrain types: 89% diesel, 4% hybrid, and 7% electric buses. The diesel buses are the most frequently used across all routes (Figure S4A). The hybrid buses are used less consistently on short routes in some months and on longer routes in other months. Electric bus services only operate on short routes due to range and charging constraints.

On average, the diesel buses traveled about 2,736 km monthly while the electric buses traveled about 1,288 km (Figure S4B). The distances traveled by hybrid buses ranged from about 1300 km in November 2021 to as high as 3400 km in September 2022. The monthly diesel consumption per vehicle for diesel buses typically exceeded 1900 liters (L) on average (Figure S4C). In comparison, hybrid buses showed a wider range of diesel usage, ranging from around 600 L to approximately 1900 L, depending on the month of the year. For example, in November 2021, the average diesel consumption was 1900 L for diesel and 1700 L for hybrid buses, while in August 2022, it was 2120 and 835 L. In terms of fuel economy, hybrid buses averaged 1.64 km/L, while diesel buses averaged 1.35 km/L. The average fuel economy is 0.77 km/kWh for electric buses (Figure S4D).

#### Optimization framework

Our optimization framework minimizes annual diesel consumption across the network over an 18-year planning horizon (2022 to 2040). We selected this timeframe to provide a realistic window for assessing fleet replacement cycles and the gradual integration of electric buses. This is in line with the legislative mandate requiring all transit agencies to achieve zero-emission status by 2040.^47^ Since our analysis is based on operational data from 2022, we selected this timeframe to provide a realistic window for assessing fleet replacement cycles and the gradual integration of electric buses. We designed the problem as a binary integer program that assigns buses by powertrain to each trip on the day of the year, *d*^∗^, with the highest number of bus trips, which we refer to as the “busiest day.” This way, we obtained fleet assignment solutions that satisfy service levels for all days of the year while simplifying the optimization problem and reducing complexity.

In the year-long APC data, *d*^∗^ corresponded to October 29, 2021, on which 2409 bus trips were served. We assumed the same bus schedule and, thus, daily trip distribution for each year of the horizon. Ultimately, 18 sequential diesel minimization problems (for the busiest day) were solved for each year in the planning horizon, thus providing the optimal fleet assignment and, consequently, powertrain composition. We used the Gurobi mathematical programming solver in Python,^48^ which employs a branch-and-cut algorithm,^49^ to solve the binary program. The optimization process took a total of 122 minutes to compute the solution across all 9 futures. The computations were performed on a Lenovo laptop with an Intel processor running at 2.83 GHz and 32 GB of RAM. The system operates on Windows 10 with Intel UHD Graphics. The model was implemented using Python and the Gurobi solver. Additional convergence plots for all nine optimization problems are provided in Figure S5, further illustrating solver performance across all futures.

The optimization problem is specified as:

(Equation 1) $$\min_{x_{p,\tau ,y}}\sum_{p\in \left\{C,H,B\right\}}\sum_{\tau \in T}\sum_{y\in Y}{\hat{D}}_{p,\tau ,y,d^{*}}\cdot x_{p,\tau ,y}$$

(Equation 2) $$s.t.\sum_{p\in P}x_{p,\tau ,y}=1\;\forall \tau \in T,y\in Y$$

(Equation 3) $$\sum_{\tau \in T}d_{\tau }x_{p,\tau ,y}-N_{p,y}r_{p}\leq 0\;\forall y\in Y,p\in P$$

(Equation 4) $$N_{B,y}-M_{y}^{cap}\leq 0\;\forall y\in Y$$

(Equation 5) $$\sum_{p\in \left\{H,B\right\}}\cos\;t_{p}\cdot \max\left[0,\left(N_{p,y}-N_{p,y-1}\right)\right]-M^{inv}\leq 0\;\forall y\in Y$$

(Equation 6) $$\sum_{p\in P}N_{p,y}-M^{flt}\leq 0\;\forall y\in Y,p\in P$$

where ${\hat{D}}_{p,\tau ,y,d^{*}}$ is the predicted diesel consumption for trip *τ* operated by a bus of powertrain type *p* in the busiest day *d*^∗^ of year *y*, *p* is the powertrain, and *τ* is a unique identifier for each trip. The decision variables *x*_*p*,*τ*,*y*_ are binary, indicating if a bus of powertrain type *p*∈{*C*,*H*,*B*}—representing diesel, hybrid, and electric buses, respectively—is assigned to operate a trip *τ* in year *y*.

The constraints considered included budget cap, charging capacity, and bus range. Constraint ((2)) requires that only one bus powertrain type is assigned to each trip, ensuring that all trips are serviced. Constraint ((3)) ensures that, for each powertrain type *p* in each year *y*, the total distance of assigned trips does not exceed the available daily operating range of the fleet, which is given by *N*_*p*,*y*_·*r*_*p*_, where *r*_*p*_ is the daily range of buses of type *p*. Our model optimizes at the powertrain-to-trip assignment level, estimating the total number of buses required for each powertrain type based on cumulative trip assignments and range limitations, rather than assigning individual buses to specific trips. When the range of electric buses prevents direct assignment to certain longer routes, the model compensates by assigning additional electric buses to collectively cover those service kilometers. This constraint allows the number of buses *N*_*p*,*y*_ to be determined endogenously by the model based on the total trip assignments. To avoid overestimating fleet sizes, we introduce an auxiliary constraint that links *N*_*p*,*y*_ to the actual number of trips assigned to powertrain *p* in year *y*, ensuring that buses are only included in the fleet if they are actively used. This approach dynamically adjusts fleet size each year while avoiding the inclusion of idle vehicles, providing a realistic representation of operational requirements. To account for deadheading and unmodeled distances between trips, we subtracted a 40 km buffer from the nominal daily range values *r*_*p*_ for all powertrain types. This adjustment reflects the model’s abstract assignment of powertrains to trips rather than exact vehicle dispatching. We calibrated the buffer to yield a fleet size and composition that closely matches PVTA’s observed fleet in the base year.

Constraint ((4)) ensures that the total number of daily charging slots required each year for electric buses in the fleet, *N*_*B*,*y*_, does not exceed the available charging capacity $M_{y}^{cap}$. We assumed that all electric buses are charged overnight, consistent with the current operational practices in transit agencies similar to PVTA. This simplifies the model by avoiding mid-day charging considerations. Constraint ((5)) ensures that new hybrid and electric bus purchases (*N*_*p*,*y*_-*N*_*p*,*y*-1_) do not exceed the annual budget cap. The parameter *cost*_*p*_ reflects the differentiated purchase cost of each powertrain, which is assumed to be 0.4*M* for diesel, 0.9*M* for hybrid, and 1.3*M* for electric buses. Constraint ((6)) ensures that the total number of buses (across all types) for each year does not exceed the maximum possible fleet size, given by *M*^*flt*^=1000.

Based on existing infrastructure and current planning until 2028 in the case study network, charging capacity is assumed to start as low as 23 buses per day in 2023, increasing gradually to 73 buses per day in 2028. From then on, we assume the capacity increases annually by 40% until the end of the planning horizon in 2040 (Figure S3). In reality, the charging capacity increases from one year to the next may be less gradual, reflecting distinct budget and upgrade periods. However, a constant annual growth rate is applied here, given the uncertainties in the timeline for such investments and for ease of computation and comparison across futures.

#### Energy modeling framework

##### Trajectory computation

We computed bus trajectories within the network using geolocated and timestamped APC data from October 2021 to September 2022. Each bus trajectory *i* consists of distance *s*_*i*,*t*_, speed *v*_*i*,*t*_, and acceleration *a*_*i*,*t*_ for a given time sequence. The distance *s*_*i*,*t*_ was calculated using the Haversine formula, which considers the Earth’s curvature through the latitude and longitude of the buses at consecutive timestamps *t*-1 and *t*.^50^ The following equations were used:

(Equation 7) $$\begin{array}{l} s_{i,t}=2r\;\sin^{-1}\sqrt{\sin^{2}\left(\frac{\varphi_{i,t}-\varphi_{i,t}}{2}\right)+\cos\;\varphi_{i,t-1}\;\cos\;\varphi_{i,t}\;\sin^{2}\left(\frac{\lambda_{i,t}-\lambda_{i,t-1}}{2}\right)} \end{array}$$

(Equation 8) $$v_{i,t}=\frac{s_{i,t}-s_{i,t-1}}{\Delta t}$$

(Equation 9) $$a_{i,t}=\frac{v_{i,t}-v_{i,t-1}}{\Delta t}$$

where *r* is the radius of the Earth; *φ*_*i*,*t*_, *φ*_*i*,*t*-1_ are the latitudes, and *λ*_*i*,*t*_, *λ*_*i*,*t*-1_ are the longitudes of vehicle *i* at consecutive timestamps; and Δ*t* is the time interval between consecutive timestamps *t* and *t*-1.

Trajectories for a selected 24-hour period for a sample bus from each powertrain in the PVTA fleet are shown in Figure S6. The cumulative distribution functions (CDFs) of these computed trajectories, corresponding to the APC data for October 2021 to September 2022, are shown in Figure S7.

To ensure that erroneous observations and otherwise inconsistent data were not included in the energy model calibration, we performed a two-step data-trimming process on the acceleration and fuel economy. We calculated fuel economy (km/L) based on the refueling data and the corresponding cumulative distances obtained through trajectory computation. In the first step of the data trimming process, we filtered the acceleration data to exclude extreme observations and retained only those within the [0.01^th^,99.99^th^] percentile interval, which corresponded to accelerations in the range [-1.67,3.89] m/s^2^. In effect, this excluded 0.02% of acceleration values at the extremes (Figure S7). In the second step, we trimmed the fuel economy data in the [5^th^,95^th^] percentile range. This mitigated discrepancies in the refueling ticket dataset, which included entries with implausibly small amounts of fuel for long distances or excessively large refueling for short distances, indicative of potential refueling data errors. For further reference, the cumulative distribution functions of fuel economies are shown in Figure S8.

We developed a vehicle-level model using powertrain-specific submodels,^43^^,^^51^^,^^52^ to predict the energy consumption of the diesel and hybrid buses in the fleet. The powertrain-specific submodels incorporate real-time dynamics such as distance traveled, vehicle speed, acceleration, passenger load, and road gradient. The process begins by computing tractive power (in kW), which is the power required to move the buses. This is then followed by a fuel consumption function, which computes the diesel consumed (L) in each time interval. Finally, we calibrated the powertrain-specific coefficients in the submodels using trajectories computed from the APC data and validated model results.

##### Tractive power function

The tractive power of vehicles refers to the power generated by the engine that is directly used for driving the vehicle forward. It accounts for air resistance, road surface resistance, and the challenges of uphill and downhill travel. It also factors in the need for additional power when the vehicle speeds up. Efficiency in converting fuel or battery power into motion and the vehicle’s weight, including its passengers, are also essential components of the tractive power computation. The instantaneous tractive power output *P*_*p*,*i*,*t*_, in kW, of a vehicle *i* of powertrain type *p* at timestamp *t*, is given by:^51^

(Equation 10) $$P_{p,i,t}=\frac{v_{i,t}}{10^{3}\eta_{d,p}}\left[\rho C_{D}C_{H_{i}}A_{p}v_{i,t}^{2}+m_{i,t}\left(g\left(C_{r1}v_{i,t}+C_{r2}+G_{i,t}\right)+1.2a_{i,t}\right)\right]$$

where *v*_*i*,*t*_ is the speed of bus *i* (m/s) at timestamp *t*, *η*_*d*,*p*_ is the driveline efficiency, *ρ* is the air density (kg/m^3^), *C*_*D*_ is the drag coefficient, $C_{H_{i}}$ is the altitude correction factor, *A*_*p*_ is the frontal area (m^2^), *m*_*i*,*t*_ is the mass of the bus, including passengers (kg), *g* is the gravitational acceleration (m/s^2^), *C*_*r*1_ and *C*_*r*2_ are the rolling resistance coefficients, *G*_*i*,*t*_ is the road grade (tangent), and *a*_*i*,*t*_ is the acceleration (m/s^2^).

##### Fuel consumption submodels

The vehicle-level energy model comprises diesel, hybrid, and electric submodels. For diesel and hybrid, we estimated the diesel consumption (L) as a quadratic function of tractive power,^51^ where the diesel *D*_*p*,*i*,*t*_ consumed by bus *i* of powertrain *p* at timestamp *t* is given by:

(Equation 11) $$D_{p,i,t}=\Delta_{t}\left[\alpha_{0,p}+\left(\alpha_{1,p}P_{p,i,t}+\alpha_{2}P_{p,i,t}^{2}\right)I\left(P_{p,i,t}\geq 0\right)\right],p\in \left\{C,H\right\}$$

where Δ_*t*_ is the time interval between timestamp *t* and timestamp *t*-1, *α*_0,*p*_, *α*_1,*p*_ and *α*_2_ are the quadratic model coefficients and *P*_*p*,*i*,*t*_ is the tractive power. The primary difference between the diesel and hybrid models lies in the calibration of the coefficients (*α*_0_ and *α*_1_), which are specific to each powertrain type.

The electric submodel,^42^^,^^43^ calculates the electrical energy *E*_*i*,*t*_ in kWh. It incorporates battery and motor efficiencies and explicitly accounts for regenerative braking in regions of negative power and deceleration. Thus:

(Equation 12) $$E_{i,t}=\frac{\Delta_{t}P_{B,i,t}}{3600\eta_{m}\eta_{b}}\left\{ \begin{array}{cc} 1, & \text{if}\;P_{B,i,t}\geq 0\\ {\left(\eta_{m}\eta_{b}\eta_{d,B}\right)}^{2}\eta_{rb}, & \text{if}\;P_{B,i,t}<0\;\text{and}\;a_{i,t}<0\\ 0, & \text{otherwise} \end{array}\right.$$

where *η*_*b*_ and *η*_*m*_ are the battery and motor efficiencies, respectively, and *η*_*rb*_ is the regenerative braking energy efficiency factor which is defined as:

(Equation 13) $$\eta_{rb}=\exp\;\left[-\gamma {\left|a_{i,t}\right|}^{-1}\right]$$

where *γ* is the regenerative braking decay parameter. To better illustrate the behavior of the regenerative braking in our model, we plot the regenerative braking energy efficiency factor *η*_*rb*_ as a function of acceleration (Figure S9). In this formulation, regenerative braking energy efficiency increases with stronger braking (higher deceleration rates). At low braking intensities (acceleration values closer to zero), the recovery factor decreases, indicating that less energy is effectively regained.

##### Calibration

We calibrated the powertrain-specific parameters of the energy submodels using refueling and recharging records collected from September 2021 to October 2022. A grid search method was applied to determine optimal values for *α*_0,*p*_, *α*_1,*p*_, *γ*, *η*_*b*_, *η*_*d*,*B*_, and *η*_*m*_. These parameters represent key coefficients for fuel and energy consumption modeling across diesel, hybrid, and electric buses. To avoid overfitting, we split the data into 80% for training and 20% for testing. We used only the training set to choose model parameters and kept the test set for final validation.

**Table:** Training and test observations for energy models

Powertrain	Training	Test	Total
Diesel	18,166	4,542	22,708
Hybrid	758	190	948
Electric	132	33	165

The optimal powertrain-specific parameters were selected to minimize the root mean squared error (RMSE) and mean absolute percentage error (MAPE) between the observed ($D_{p,i,r_{i}}$) and predicted (${\hat{D}}_{p,i,r_{i}}$) amount of diesel consumed by each vehicle between consecutive refuelings, where

(Equation 14) $${\hat{D}}_{p,i,r_{i}}=\sum_{t\in r_{i}}{\hat{D}}_{p,i,t}$$

and $r_{i}\in \left\{0,1,\ldots ,N_{r_{i}}\right\}$ is the refueling interval index for each bus *i* (buses are refueled at various times of day, with the average interval being 24 hours). The RMSE is then given as

(Equation 15) $$RMSE_{p}=\sqrt{\frac{1}{N_{p}N_{r_{i}}}\sum_{i=1}^{N_{p}}\sum_{r_{i}=1}^{N_{r_{i}}}{\left(D_{p,i,r_{i}}-{\hat{D}}_{p,i,r_{i}}\right)}^{2}}$$

and the MAPE as

(Equation 16) $$MAPE_{p}=\frac{100\%}{N_{p}}\sum_{n=1}^{N_{p}}\sum_{r_{i}=1}^{N_{r_{i}}}\left|\frac{D_{p,i,r_{i}}-{\hat{D}}_{p,i,r_{i}}}{D_{p,i,r_{i}}}\right|$$

where *N*_*p*_ is the number of vehicles of powertrain type *p*∈{*C*,*H*}. For *p*∈{*B*}, we considered energy consumption (*E*_*i*,*t*_) instead of diesel consumption.

The optimal parameter values obtained through calibration, as well as their respective search ranges and sample sizes are summarized below. These optimized values were used in all subsequent energy consumption estimates throughout the study.

**Table:** Calibrated parameter values, search ranges, and sample sizes for energy submodels

Powertrain	Parameter	Description	Optimal value	Search range	Samples
Diesel	${\hat{\alpha }}_{0,C}$	Constant term	1.73·10^−3^	[10^−4^,10^−2^]	100
	${\hat{\alpha }}_{1,C}$	Linear term	1.19·10^−4^	[10^−5^,10^−3^]	100
Hybrid	${\hat{\alpha }}_{0,H}$	Constant term	1.32·10^−3^	[10^−4^,10^−2^]	100
	${\hat{\alpha }}_{1,H}$	Linear term	8.9·10^−5^	[10^−5^,10^−3^]	100
Electric	*Γ*	Regenerative braking decay	8.89·10^−3^	[10^−8^,10^−2^]	100
	*η*_*b*_	Battery efficiency	9·10^−1^	[0.70,0.99]	10
	*η*_*d*,*B*_	Driveline efficiency	9.9·10^−1^	[0.70,0.99]	10
	*η*_*m*_	Motor efficiency	8.7·10^−1^	[0.70,0.99]	10

##### Validation

We validated the calibrated energy submodels using metrics including RMSE, MAPE, Mean Absolute Error (MAE), and Median Absolute Percentage Error (MdAPE) computed on the test set. All metrics were computed at the vehicle refueling interval-level ($D_{p,i,r_{i}}$) for diesel and hybrid (the resolution used for calibration), at the daily level for electric, and the weekly system level. The observed weekly system-level diesel consumption *D*_*p*,*w*_ for powertrain *p* and week *w* is given by:

(Equation 17) $$D_{p,w}=\sum_{i=1}^{N_{p}}\sum_{t\in w}D_{i,p,t}$$

where *D*_*i*,*p*,*t*_ is the diesel consumption of bus *i* at timestamp *t* and *N*_*p*_ is the number of buses of powertrain *p* in the fleet. The performance metrics are summarized in Table S1. At the vehicle level, the MAPE was 22% for diesel, 24% for hybrid, and 26% for electric buses. At the system level, after aggregating weekly fuel and energy consumption, the errors reduced significantly to 6% for diesel, 8% for hybrid, and 13% for electric buses. These results indicate that, while vehicle-level predictions exhibit some variability, particularly for electric buses due to their limited operational history and smaller fleet size, the model’s performance improves when aggregated at the system level. Our calibrated model performs vastly better (MAPE values of 22%, 24%, and 26% for diesel, hybrid, and electric, respectively) compared to the simplistic estimation based on average fuel economy (MAPE values of 67% for diesel, 72% for hybrid, and 46% for electric; see Table S2).

##### Scaling up diesel consumption

The optimal diesel consumption $D_{y,d^{*}}$ on the busiest day *d*^∗^ in year *y* is computed as:

(Equation 18) $$D_{y,d^{*}}^{*}=\sum_{p\in \left\{C,H,B\right\}}\sum_{\tau \in T}D_{p,\tau ,y,d^{*}}$$

To scale up $D_{y,d^{*}}^{*}$ for the entire year $D_{y}^{*}$, we computed factors *f*_*d*_, representing the proportion of predicted diesel consumption on each day *d* relative to the busiest day, *d*^∗^. Figure S10 shows the daily number of bus trips in the dataset and the corresponding scaling factor *f*_*d*_, based on relative with the red dashed line at $f_{d^{*}}=1$. For simplicity, we keep *f*_*d*_ constant across the same day in each year of the optimization across all the futures. Given that either the number of hybrid or electric buses is increasing annually in all the futures except *Status Quo*, this approximation may overestimate the cumulative annual diesel consumption. However, we consider this adequate for our analyses, as it provides the “best worst-case” estimate of diesel consumption in each future.

By calculating *f*_*d*_ as the ratio of the energy consumption of each day to that of the busiest day, we accounted for daily operational variations. These factors were then used to estimate the annual diesel consumption as follows:

(Equation 19) $$D_{y}^{*}=\sum_{d\in y}f_{d}D_{y,d^{*}}^{*}$$

##### Emissions computation

Emissions calculations are based on the outputs of our energy model, which incorporates detailed operational data, including vehicle distance, speed, acceleration, road grade, and dynamic mass. We computed annual GHG emissions (CO _2_ e), using a well-to-wheel (WTW) approach that accounts for both downstream (tank-to-wheel, TTW) and upstream (well-to-tank, WTT) emissions associated with each powertrain. We used the optimized assignments of vehicle mileage to the bus powertrain type, which are the outputs from our energy model. To ensure consistency across all pollutants, we converted all emission factors to a per-km basis and applied separate factors for diesel, hybrid, and electric buses, accounting for differences in fuel efficiency and energy use.

For diesel and hybrid buses, we calculated TTW CO_2_ emissions by dividing the standard emission factor of 2,698 g/L of diesel^53^ by the fuel economy, *eco*_*p*_, of each powertrain (1.64 km/L for hybrid and 1.35 km/L for diesel as per the output of the energy model). For non-CO_2_ pollutants, we applied methane (CH_4_) and nitrous oxide (N_2_O) emission rates of 0.0059 g/km and 0.0268 g/km, respectively.^53^ These rates were scaled for hybrid buses based on their higher fuel economy relative to diesel buses. All non-CO_2_ emissions were converted to CO_2_ e using global warming potential (GWP) factors of 28 gCO_2_ e/gCH_4_ and 265 gCO_2_ e/gN_2_O.^54^ Thus, the TTW emissions component (measured in gCO2_e_) is given by:

(Equation 20) $$M_{y}^{\text{TTW}}=\sum_{p\in \left\{C,H\right\}}VKT_{p,y}\cdot \left[\frac{2,698}{eco_{p}}+\frac{eco_{p}}{eco_{C}}\left(0.0059\cdot 28+0.0268\cdot 265\right)\right]$$

where, *VKT*_*p*,*y*_ is the total annual distance (km) traveled by powertrain type *p* in year *y*, *eco*_*p*_ is the fuel economy of powertrain *p*, and *eco*_*C*_ is the baseline diesel fuel economy used to normalize non-CO_2_ emissions.

In addition to tailpipe emissions, we accounted for upstream WTT emissions associated with diesel fuel production and distribution, estimated at 536 gCO_2_ e/L of diesel.^55^ This was likewise scaled by the fuel economy of each powertrain to express WTT contributions on a per-km basis. For electric buses, lifecycle emissions were calculated by multiplying the predicted energy consumption per kilometer (kWh/km) by regional electricity emissions factors for CO_2_, CH_4_, and N_2_ O. We used values of 243.41 gCO_2_/MWh, 28.58 gCH_4_/MWh, and 3.63 gN_2_ O/MWh, from the eGRID Northeast region.^53^ We then converted these emission factors to CO_2_ e using global warming potential (GWP) factors of 28 gCO_2_ e/gCH_4_ and 265 gCO_2_ e/gN_2_O.^54^ Since electric buses do not emit tailpipe GHGs, we attributed all emissions from electricity generation to the WTT phase. Thus, the WTT component (measured in gCO2_e_) is given by:

(Equation 21) $$M_{y}^{\text{WTT}}=\sum_{p\in \left\{C,H\right\}}VKT_{p,y}\cdot \frac{536}{eco_{p}}+VKT_{B,y}\cdot \left(\frac{10^{-3}}{eco_{B}}\cdot \left[243.41+\left(28.58\cdot 28\right)+\left(3.63\cdot 265\right)\right]\right)$$

where *eco*_*B*_ is the average energy efficiency of electric buses in km/kWh.

The cumulative WTW GHG emissions (measured in gCO2_e_) over the planning horizon were thus computed as the sum of TTW and WTT emissions for each powertrain. Thus, for each year *y*, we define total lifecycle GHG emissions as:

(Equation 22) $$M_{y}^{\text{WTW}}=M_{y}^{\text{TTW}}+M_{y}^{\text{WTT}}$$

#### Experimental design

The goal of the experimental design is to minimize diesel consumption over an 18-year horizon under various fleet transition strategies, with constraints on budget, charging, and bus range. We considered four fleet transition strategies and three budget scenarios. From these, we obtained ten futures, each representing a budget scenario evaluated under a given purchase strategy. In each future, except the *Status Quo*, the diesel consumption was minimized across the 18-year horizon, resulting in decision variables for trip assignment by powertrain type. Each future was then analyzed for impacts on fleet composition, diesel consumption, emissions, and costs.

##### Strategies

We define four strategies that explore various approaches to transitioning the bus fleet toward reduced or zero emissions. These strategies are: *Status Quo*, *Hybrid*, *Hybrid+Electric*, and *Electric*. Key descriptions are provided in Table 1.

In the *Status Quo*, the current fleet composition is maintained without pursuing additional hybridization or electrification when purchasing buses during the horizon period. When the existing fleet reaches its useful life, they are replaced with the same powertrain, thus preserving the current fleet composition or *Status Quo*. In the case of the PVTA network, this preserves the baseline fleet composition of 89% diesel, 4% hybrid, and 7% electric buses until 2040. No additional constraint is required for this future.

Under the *Hybrid* purchase strategy, diesel buses are replaced with hybrid, and thus, all newly purchased buses are hybrid. This is achieved by imposing a constraint that prevents the number of electric buses from increasing relative to the previous year.

(Equation 23) $$N_{B,y}\leq N_{B,y-1}$$

The *Hybrid+Electric* purchase strategy allows newly purchased fleets to be either hybrid or electric powertrains. The resulting fleet distribution is obtained as the diesel-minimizing combination, given the range constraints, budgetary constraints, and others defined in (Equation 2), (Equation 3), (Equation 4), (Equation 5), (Equation 6). No additional constraint is needed for this purchase strategy. The *Hybrid+Electric* strategy is the non-constrained approach where the transit agency can purchase both hybrid and electric buses to maximize emissions reductions.

In the *Electric* purchase strategy, all future fleet acquisitions are electric powertrains. This purchase strategy includes fixed electric charging capacity and electric bus range constraints. This purchase strategy is operationalized by ensuring that the number of hybrid buses does not increase relative to the previous year. The additional constraint is given by:

(Equation 24) $$N_{H,y}\leq N_{H,y-1}$$

##### Budget scenarios

We designed three budget scenarios, each of which imposes a limit on the maximum amount *M*^*inv*^ that can be spent each year on new bus purchases. For reference, we note that purchase prices assumed for a bus of a given powertrain type are: $0.4M (diesel), $0.9M (hybrid), and $1.3M (electric). We define a *future* as the realization of a budget scenario under a given fleet transition purchase strategy. Thus, from the fleet purchase strategies (*Status Quo*, *Hybrid*, *Hybrid+Electric*, *Electric*) and budget scenarios (*lo-cap*, *mid-cap*, *hi-cap*), 10 futures were evaluated (see Table 1). The baseline *Status Quo* purchase strategy was evaluated only under the *lo-cap* scenario, as the required budget for that purchase strategy was within the $10M annual cap. Hereafter, we refer to the *lo-cap Status Quo* future as simply *Status Quo*. In the optimization model, each future is associated with specific constraints that govern new fleet purchases and the budget. These constraints ensure that the optimization (diesel minimization) results are tailored to the strategic and financial realities of each future, providing a range of feasible pathways.

##### Costs

While reductions in diesel consumption and emissions are important metrics used to evaluate and compare the optimized futures, the costs required to achieve environmental savings are also an important consideration, especially from an investment and decision-making perspective. We considered three different costs: fleet investment, operating costs, and maintenance costs. We calculated fleet investment costs based on the assumed current prices of $0.4M, $0.9M, and $1.3M for a single diesel, hybrid, and electric bus, respectively.

For operating costs, which include both fuel and energy consumption, we relied on the output from the developed energy models. The cost of diesel fuel was set at $0.95/L,^56^ while the cost of electricity was $0.11/kWh.^57^ These rates, combined with the energy consumption predicted by the models, were used to compute the cumulative operating costs. For maintenance costs, we factored in both the per-km and per-year maintenance costs for each powertrain type.^58^ To reflect the time value of money, we applied a 4% discount rate to all costs, including fleet purchase, operating, and maintenance, across the planning horizon. This allowed for consistent cost comparison across years and ensured that future expenditures were appropriately weighted in the evaluation of each future.

**Table:** Summary of operating and maintenance costs

Metric	Diesel	Hybrid	Electric
Operating costs (fuel/energy)	$0.95/L	$0.95/L	$0.11/kWh
Distance-based maintenance costs	$0.677/km	$0.683/km	$1.658/km
Vehicle-based annual maintenance costs	$2,142/bus/year	$2,142/bus/year	$4,643/bus/year

#### Sensitivity analysis

To evaluate the robustness of the developed fleet electrification optimization framework, we conducted a sensitivity analysis on two key parameters, including charging capacity and electric bus range. These two parameters play a critical role in shaping the feasibility of electrification. Charging infrastructure directly affects the ability to transition to electric buses, and bus range determines operational flexibility. Taking into account the uncertainties in infrastructure expansion, we evaluated assumptions in which the annual charging capacity decreases from 50% to increases by 100% beyond the baseline estimates. Battery electric bus range also varies due to factors such as weather conditions and terrain grade. To evaluate the impact of these variations, we analyzed assumptions where the electric bus range is reduced by 50% to increased by 100% relative to the base case. Thus, we quantified the impact of both charging infrastructure and range uncertainty on optimal fleet composition and diesel consumption.

### Quantification and statistical analysis

All statistical analyses were performed in Python using custom scripts and the Gurobi Optimizer. The calibration and validation of the model were based on performance metrics including the root mean squared error (RMSE), mean absolute error (MAE), mean absolute percentage error (MAPE), and median absolute percentage error (MdAPE).
