# Supplementary material for: Harnessing hybrid buses in the near term leads to faster transit decarbonization
Source: iScience. 2025 Sep 13;28(10):113567. doi: 10.1016/j.isci.2025.113567 (PMC12514564; doi:10.1016/j.isci.2025.113567)
Supplement: Document S1. Figures S1–S10 and Tables S1 and S2 [file mmc1.pdf]

iScience, Volume *28*

Supplemental information

Harnessing hybrid buses in the near term leads to faster transit decarbonization

Mahsa Arabi, Tolu Oke, Erin Baker, and Jimi Oke

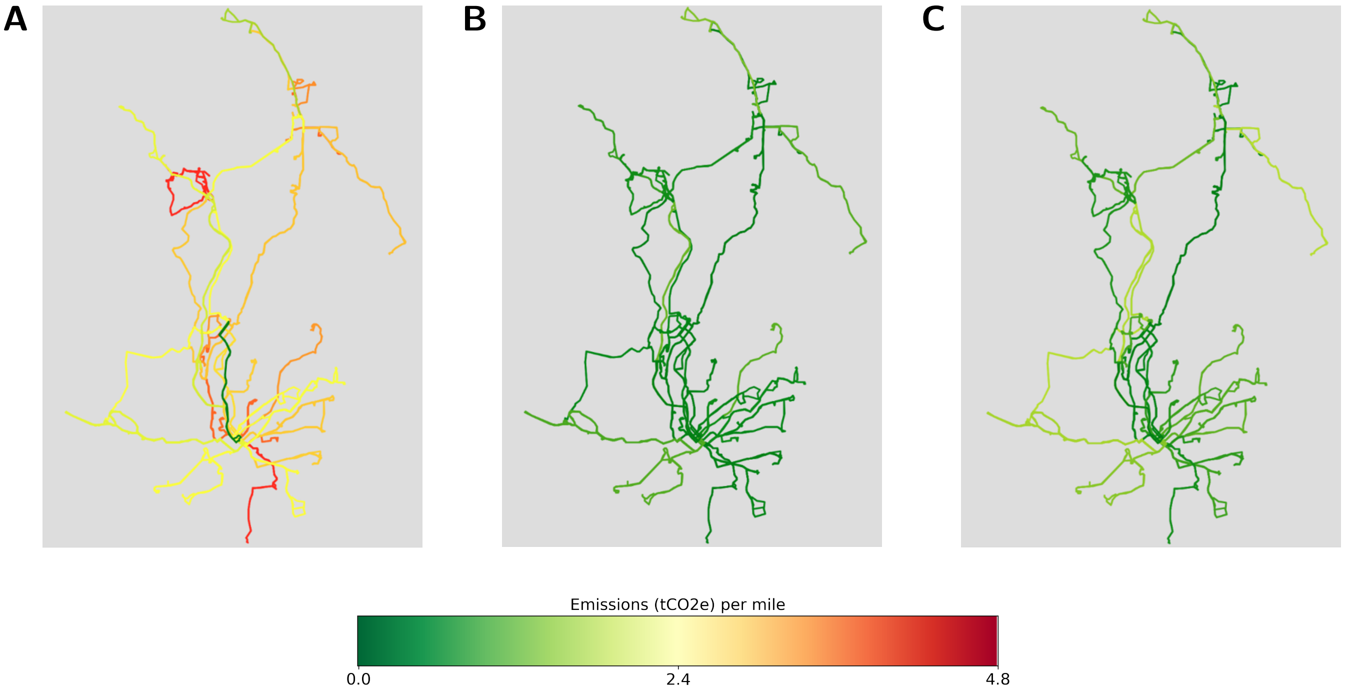

Figure S1: Route-level optimized yearly emissions (tCO_2e) per-km of the year 2040 for (A) *Status Quo*, (B) *hi-cap Hybrid+Electric*, and (C) *hi-cap Electric*

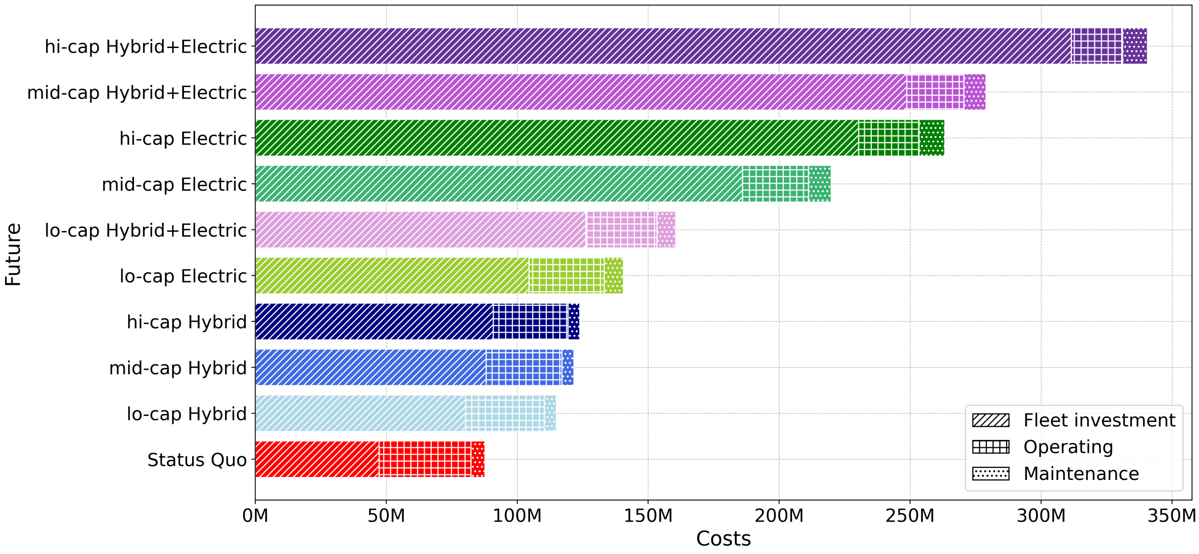

Figure S2: Cumulative cost requirements, including fleet investment, maintenance, and operating costs for simulated futures (2022-2040).

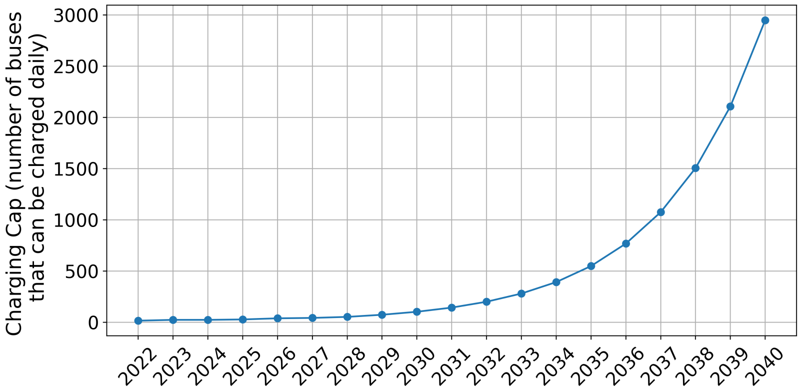

Figure S3: Charging capacity (number of buses that can be charged daily) during planning horizon

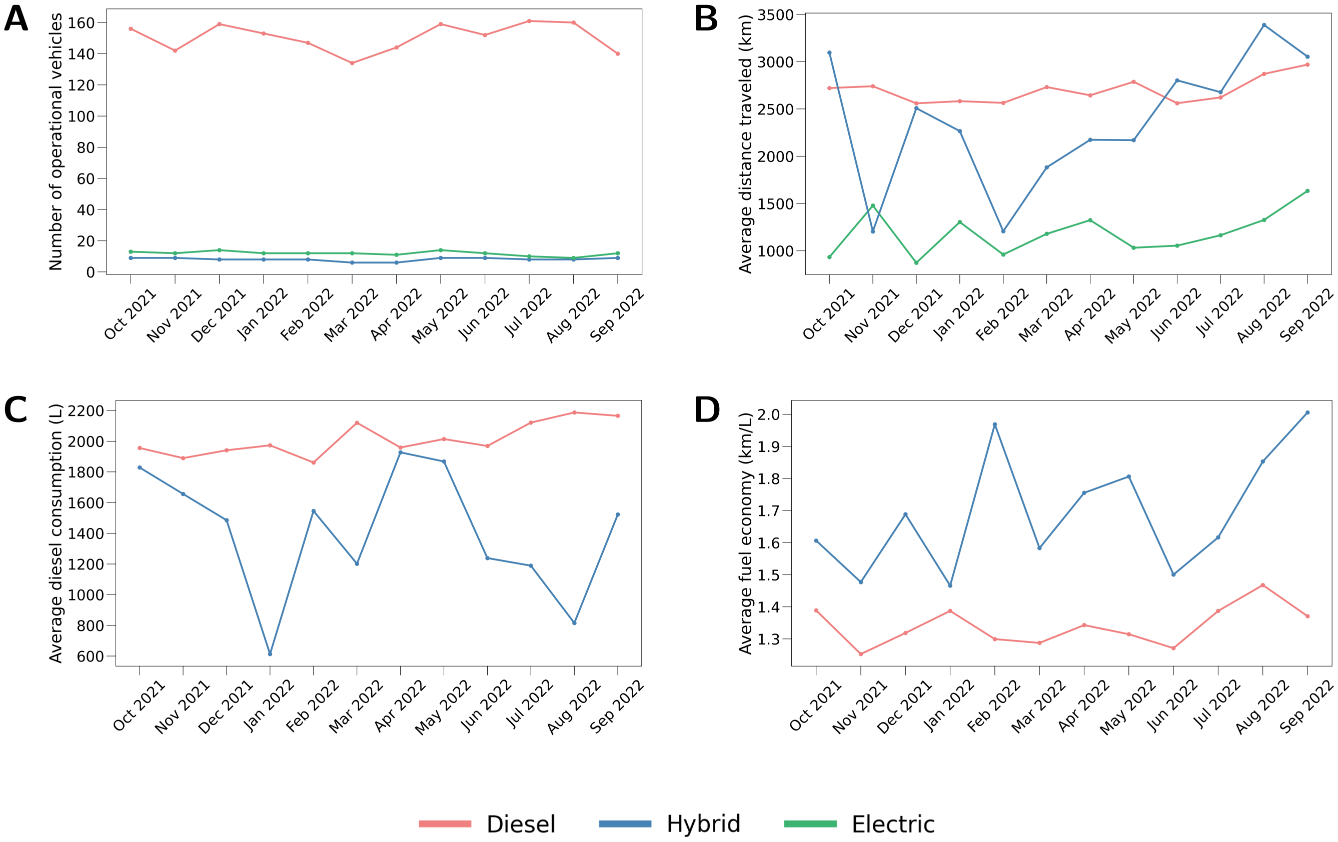

Figure S4: Summary of operational fleet data categorized by powertrain—diesel, hybrid, and electric—from October 2021 to September 2022: (A) The number of operational vehicles, (B) Monthly average distance traveled per bus, (C) Monthly average diesel consumption per bus, and (D) Average fuel economy (km/L) for diesel and hybrid buses.

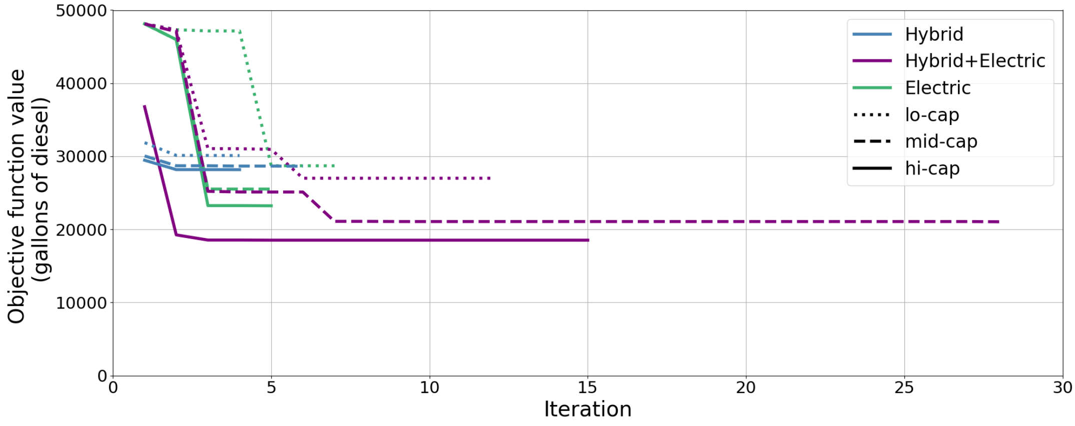

Figure S5: Optimization convergence plots for each future

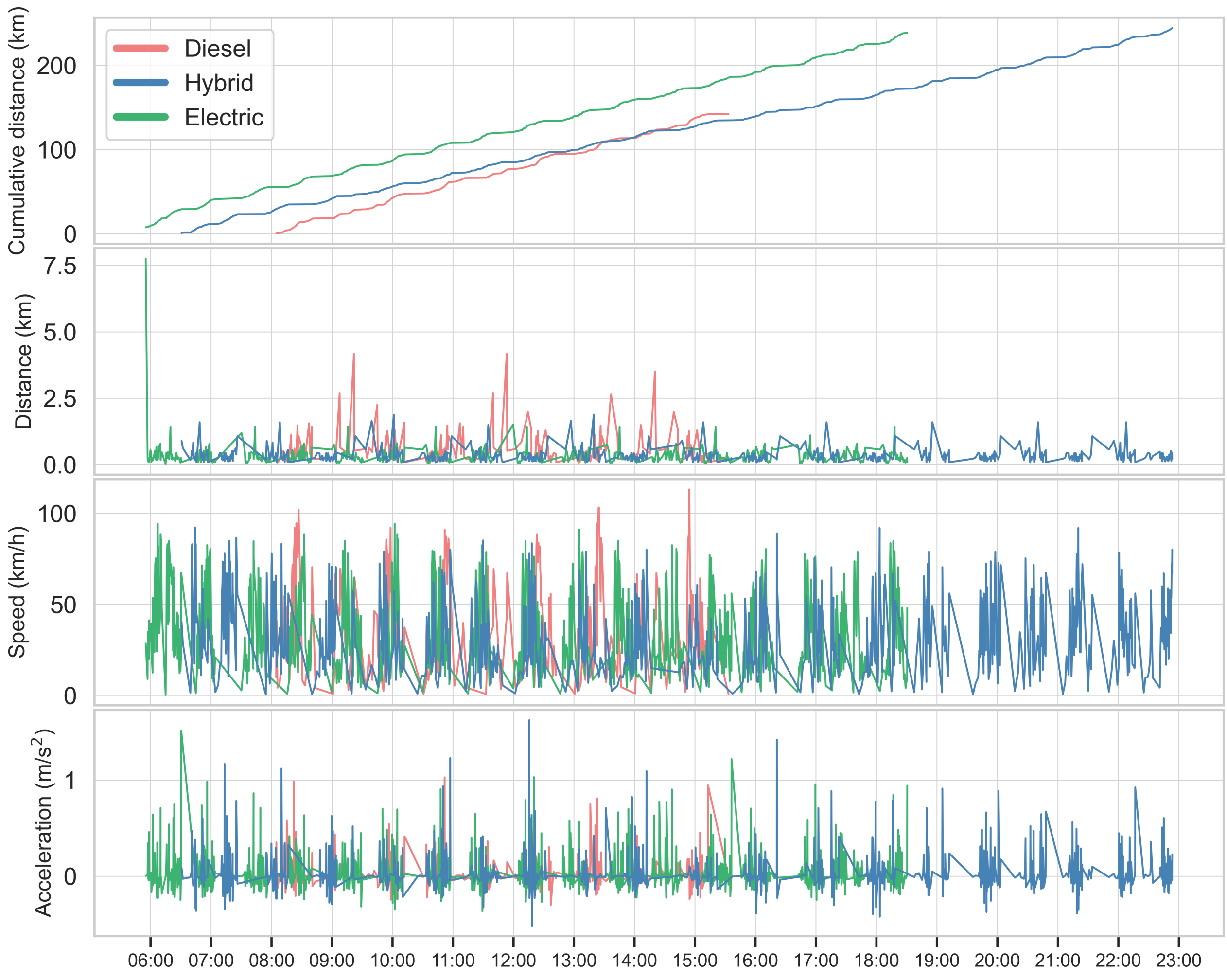

Figure S6: Time series of trajectories (cumulative distance, speed, and acceleration) for one sample bus from each powertrain in the PVTA fleet over a 24-hour period (5:00 a.m., August 23, 2022, to 5:00 a.m., August 24, 2022).

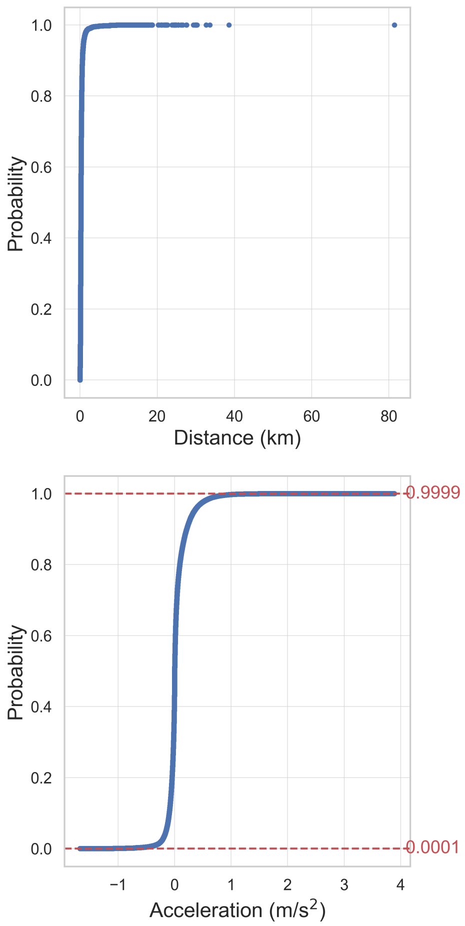

Figure S7: Cumulative distribution function (CDF) of trajectories: distance, speed, acceleration, and time intervals in timestamps from October 2021 to September 2022.

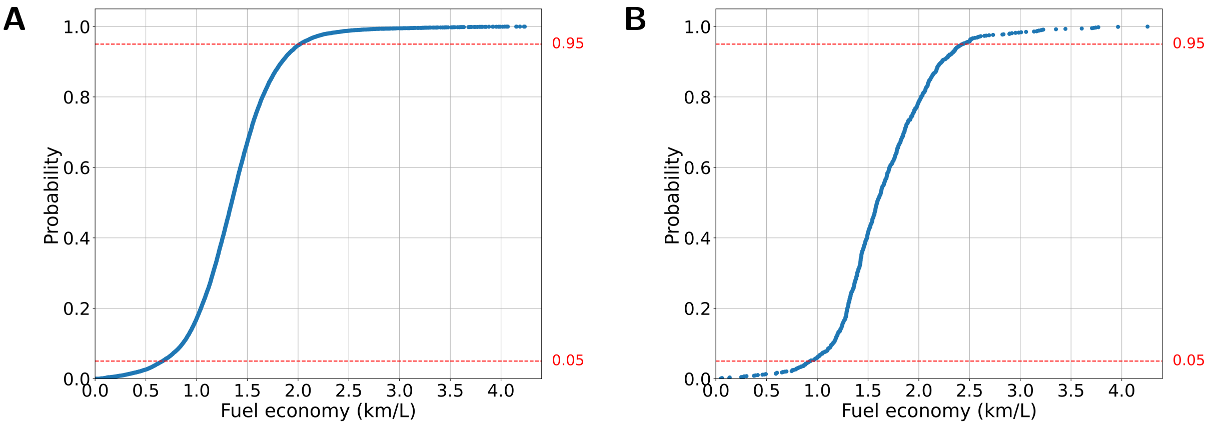

Figure S8: Cumulative distribution function (CDF) of actual fuel economy for (A) diesel and (B) hybrid buses

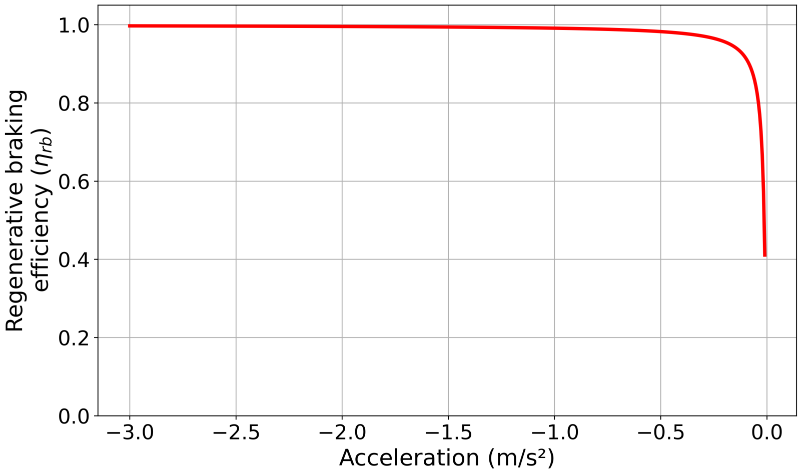

Figure S9: Effect of deceleration on the regenerative braking energy efficiency (η_{rb}). Efficiency increases with braking intensity, following the exponential decay term in Equation 12.

Table S1: Model performance metrics of the test set (SI units)

	Metric	Diesel	Hybrid	Electric
**Vehicle-level**	RMSE	32.21 L	34.00 L	51.75 kWh
	MAPE	22%	24%	26%
	MAE	24.79 L	24.61 L	39.3 kWh
	MdAPE	18%	16%	27%
**System-level**	RMSE	3,897 L	241.85 L	164 kWh
	MAPE	6%	8%	13%
	MAE	3,422 L	176.40 L	138 kWh
	MdAPE	7%	5%	14%

Table S2: Comparison of vehicle-level model MAPEs on the test set between the proposed model and a model based on average consumption rates. Fuel economy rates of 2.05 km/L for diesel, 2.48 km/L for hybrid, and 0.48 km/kWh for electric were applied.

Model	Diesel	Hybrid	Electric
Calibrated energy model	22%	24%	26%
Average consumption model	67%	72%	46%

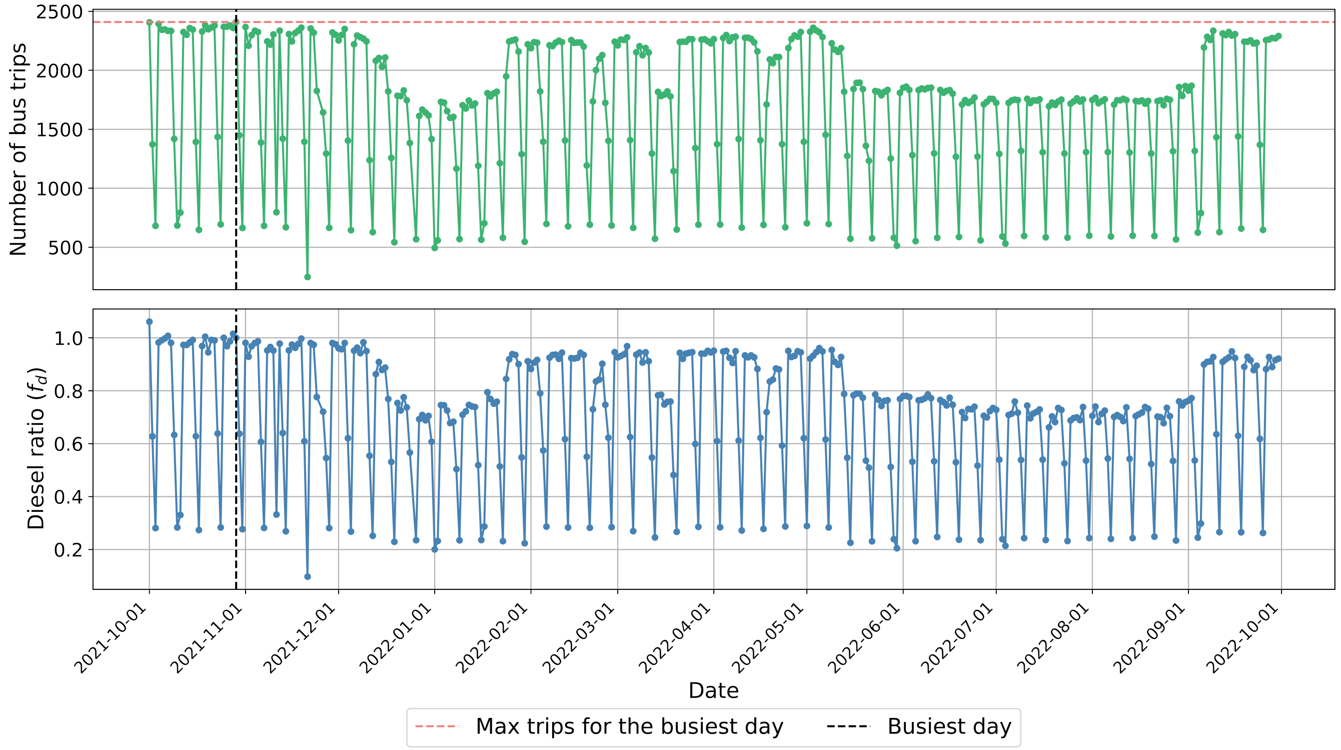

Figure S10: Number of bus trips and scaling factor f_d for extrapolating yearly energy consumption. The busiest day d^*, based on the number of bus trips, is indicated by the black dashed line, while the red dashed line highlights the maximum number of trips (2409) on day d^*.
